# Draft Genome Sequence of *Lysinimicrobium mangrovi* NBRC 105856^T^, Isolated from the Rhizosphere of a Mangrove

**DOI:** 10.1128/genomeA.01131-14

**Published:** 2014-11-06

**Authors:** Moriyuki Hamada, Natsuko Ichikawa, Akio Oguchi, Nobuyuki Fujita

**Affiliations:** a Biological Resource Center, National Institute of Technology and Evaluation (NBRC), Chiba, Japan; b NBRC, Tokyo, Japan

## Abstract

Here, we report the draft genome sequence of the only species of the genus *Lysinimicrobium*, *Lysinimicrobium mangrovi* NBRC 105856^T^, isolated from the rhizosphere of a mangrove. The first genomic sequence of this genus and species presented here will facilitate taxonomical, ecological, and functional studies of this rare actinobacterial group.

## GENOME ANNOUNCEMENT

Members of the suborder *Micrococcineae* are widely distributed in both natural and artificial habitats, e.g., in soils, plants, sediments, animals, clinical specimens, foods, and dairy products, and are known as a taxon containing many industrially useful strains (1). The genus *Lysinimicrobium*, within the family *Demequinaceae* and the suborder *Micrococcineae*, currently contains only one species, *Lysinimicrobium mangrovi* (2). Cells of this species are Gram stain positive, nonmotile, and non-endospore forming and exhibit a rod-coccus growth cycle. The peptidoglycan is of the A4α type (3), and the predominant menaquinone is demethylmenaquinone DMK-9(H_4_). Strain NBRC 105856^T^, which is the type strain of *L. mangrovi*, was isolated from a soil sample that had been collected from the rhizosphere of a mangrove (*Bruguiera gymnorhiza*) growing on Iriomote Island, Okinawa, Japan. So far, it is the only known strain belonging to the genus *Lysinimicrobium*. To reveal genomic features of this rare actinobacterial strain, we performed whole-genome shotgun sequencing.

The genomic DNA of strain NBRC 105856^T^ was extracted and purified from cells directly harvested from the culture available at the NBRC culture collection using the EZ1 DNA tissue kit and EZ1 advanced instruments (Qiagen). The whole genome of strain NBRC 105856^T^ was analyzed by using paired-end sequencing with MiSeq (Illumina) (299.8 Mbp, 101-fold coverage). These reads were assembled using the Newbler v. 2.6 software and subsequently finished using the GenoFinisher software (4), which led to 29 scaffolds of >2,000 bp each. The total length of the genome was 2,968,736 bp. The calculated G+C content of the genome was 71.79 mol%. Coding sequences were predicted by Prodigal (5). There are a total of 2,766 protein-coding genes and 47 tRNA features in the annotated genome sequence.

This is the first draft genome sequence for not only the genus *Lysinimicrobium* but also the family *Demequinaceae*, which will provide a foundation for further phylogenetic, taxonomic, comparative genomic, metagenomic, and functional studies of this genus and related taxa. In fact, since we recently isolated many novel strains which are related to the genus *Lysinimicrobium*, we will perform phylogenetic analyses, including *in silico* DNA-DNA hybridization, for the classification of isolated strains using the draft genome sequence of *L. mangrovi* NBRC 105856^T^ as a reference. Detailed reports of these analyses will be included in a future publication.

### Nucleotide sequence accession numbers.

This whole-genome shotgun project has been deposited in DDBJ/ENA/GenBank under the accession no. BBLU00000000. The version described in this paper is the first version, BBLU01000000.
